# Neoadjuvant immunotherapy plus chemotherapy for squamous cell carcinoma of the paranasal sinus: a case report

**DOI:** 10.3389/fonc.2024.1462993

**Published:** 2024-12-24

**Authors:** Ying Sun, Guanghui Yang, Ruijie Sun, Fangli Cao

**Affiliations:** ^1^ Department of Medical Oncology, Qilu Hospital of Shandong University (Qingdao), Qingdao, Shandong, China; ^2^ Department of Otolaryngology, Qilu Hospital of Shandong University (Qingdao), Qingdao, Shandong, China

**Keywords:** neoadjuvant, immunotherapy, chemotherapy, head and neck squamous cell carcinoma, major pathological response

## Abstract

**Background:**

Immune checkpoint inhibitors (ICIs) such as pembrolizumab and nivolumab are recommended as first-line therapies for recurrent and metastatic head and neck squamous cell carcinoma (HNSCC). However, their efficacy in neoadjuvant therapy remains uncertain.

**Case presentation:**

We report the case of a 68-year-old male diagnosed with HNSCC who received neoadjuvant nivolumab (anti-PD-1 inhibitor) plus nab-paclitaxel and carboplatin. Biomarkerswere assessed by immunohistochemistry, and apoptosis-related molecules were analyzed via Western blotting. The patient achieved significant tumor regression and major pathological response (MPR) without severe adverse events. Post-treatment analyses revealed PD-L1 expression increased from 30% to 50% in tumor cells, CD8+ lymphocyte infiltration significantly improved, and Ki-67 expression was markedly reduced.

**Conclusions:**

This case highlights the potential of combining ICIs with chemotherapy in neoadjuvant settings for HNSCC, providing mechanistic insights and clinical evidence for this emerging approach. Further studies are needed to establish the optimal neoadjuvant treatment regimen and identify patient populations most likely to benefit.

## Introduction

Head and neck squamous cell carcinoma (HNSCC) is a significant global health burden, accounting for more than 800,000 new cases in 2020 and causing 400,000 deaths annually (1). Despite advances in surgery and chemoradiotherapy, approximately 60% of patients present with locally advanced disease at diagnosis, and nearly half experience relapse within two years (2). This highlights an urgent need for more effective treatment strategies.

The standard neoadjuvant regimen, TPF (cisplatin + paclitaxel + 5-fluorouracil), has shown limited success in improving prognosis for all head and neck tumors (3, 4). Recently, growing evidence has demonstrated the promise of neoadjuvant immune checkpoint inhibitors (ICIs) across various malignancies. ICIs have achieved favorable pathological response rates (PCR) by harnessing the immune system’s ability to target and eliminate cancer cells (5–7). For instance, the Checkmate 816 study highlighted the benefits of combining immunotherapy with chemotherapy in the neoadjuvant treatment of lung cancer, achieving significant clinical benefits, including stage reduction and improved R0 resection rates, with a 13.6% increase in PCR compared to chemotherapy alone (6).

In the context of recurrent and metastatic HNSCC, ICIs have demonstrated survival benefits and are now integral to first-line treatment strategies (8–10). Key studies such as Checkmate-141, Keynote-040, and Keynote-048 have driven a paradigm shift in the treatment of relapsed/metastatic HNSCC, paving the way for immunotherapy to play a transformative role in earlier disease stages (8, 11–13). These findings suggest that neoadjuvant immunotherapy, particularly in combination with chemotherapy, could offer novel therapeutic avenues for patients with unresectable HNSCC.

In this case report, we describe an HNSCC patient who received neoadjuvant immunochemotherapy (nivolumab plus Nab-paclitaxel/carboplatin), resulting in a major pathological response (MPR). This case highlights the potential of combined immunotherapy and chemotherapy to achieve significant clinical benefits in HNSCC and underscores the need for further exploration of this treatment approach.

## Method

Clinical data were collected from a patient diagnosed with ethmoid sinus carcinoma, treated at the Department of Medical Oncology, Qilu Hospital (Qingdao, China). The inclusion criteria for this study included patients diagnosed with locally advanced, non-metastatic squamous cell carcinoma of the head and neck (HNSCC) (cT4N0M0), confirmed by imaging and pathological examination. Patients were required to have adequate organ function and Eastern Cooperative Oncology Group (ECOG) performance status ≤ 1. Exclusion criteria included prior exposure to immunotherapy, presence of distant metastases, severe comorbidities contraindicating immunotherapy or chemotherapy, or inability to provide informed consent. Tumor biomarkers, including PD-L1, Ki67, CD8, and IFN-γ, were evaluated through immunohistochemistry (IHC) on baseline and postoperative samples. For PD-L1 detection, SP263 staining was utilized following standard protocols.

Protein expression analysis was performed using Western blotting, as described in previous studies. Antibodies targeting Caspase-7, Caspase-3, Bcl-2, and Bax were obtained from Proteintech and diluted at ratios ranging from 1:800 to 1:2000. GAPDH was used as a loading control to ensure consistent protein quantification.

During follow-up, craniocerebral magnetic resonance imaging (MRI) was conducted every three months for the first two years to monitor disease progression. Treatment-related toxicities were assessed in accordance with the Common Terminology Criteria for Adverse Events (CTCAE), version 4.0.

Ethical approval for this study was obtained from the Ethics Committee of Qilu Hospital (Qingdao, China) under the registration number KYLL-2023083. Written informed consent was obtained from the patient before participation.

## Results

In January 2022, a 68-year-old Asian male worker presented to our hospital with a two-month history of headache, diplopia, and restricted eye movement. His Eastern Cooperative Oncology Group (ECOG) Performance Status was 1. The patient had a 10-year history of hypertension, controlled with oral ACE inhibitors, and a 20-year history of diabetes, managed with insulin. He also reported a 30-year smoking history (approximately five cigarettes per day) and a 30-year history of alcohol consumption (about 100 g per day).

Pre-treatment magnetic resonance imaging (MRI) revealed an occupying lesion in the ethmoid sinus, involving the frontal bone, sphenoid bone, left orbit, and anterior cranial fossa meninges. The lesion encased the left superior rectus muscle, superior oblique muscle, and optic nerve. A nasopharyngeal biopsy confirmed moderately differentiated papillary squamous cell carcinoma (SCC, cT4N0M0). Immunohistochemistry showed CK5/6(+), p63(+), p40(+), EGFR(+), Ki-67 (+50%), and p16(-). *In situ* hybridization demonstrated EBER(-), while PD-L1 expression was 30% in tumor cells (TC) and 10% in immune cells (IC).

Given the extensive tumor burden and the patient’s severe symptoms (e.g., headache, diplopia, and restricted eye movement), organ preservation was a high priority for the patient and his family. Considering the patient’s advanced age and inability to tolerate an aggressive TPF regimen, we opted for a combination of nivolumab and chemotherapy based on evidence suggesting improved objective response rates (ORR) with this approach in other cancer types. On February 16, 2022, the patient began treatment with nivolumab (360 mg on day 1), albumin-bound paclitaxel (125 mg/m² on days 1 and 8), and carboplatin (AUC = 5 on day 1), administered every three weeks for three cycles.

**Figure 1:**
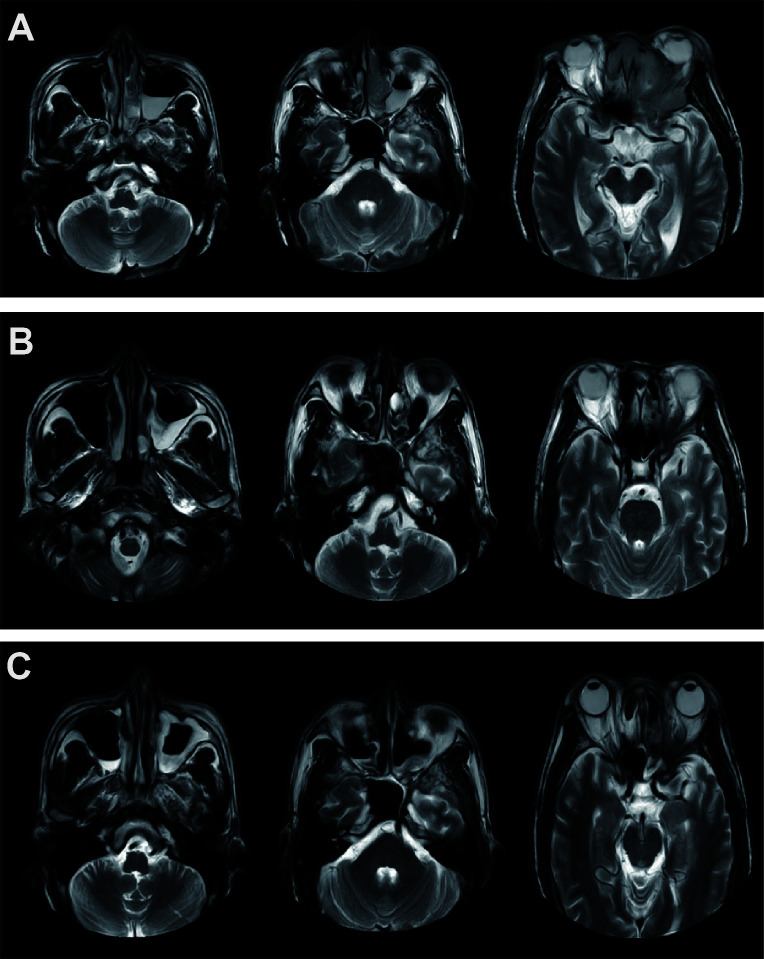
Imaging examinations of the patient during treatment. **(A)** MRI of the primary tumor before treatment. **(B)** MRI after three cycles of neoadjuvant therapy with nivolumab. **(C)** Imaging results following neoadjuvant therapy, surgery, and concurrent chemoradiotherapy.

Following three cycles of immunochemotherapy, MRI demonstrated significant tumor shrinkage (**Figure 1**). In April 2022, the patient underwent left whole-group sinus opening, bilateral middle turbinectomy, and left sinus tumor composite resection via nasal endoscopy. Postoperative pathological analysis revealed only well-differentiated SCC scattered in the left orbital wall, with no residual tumor cells in the left ethmoid sinus, frontal sinus, or nasal cavity.

Subsequently, the patient received concurrent chemoradiotherapy because only major pathological response (MPR) rather than pathological complete response (PCR) was achieved. Intensity-modulated radiotherapy (IMRT) was delivered with the following parameters: 60.06 Gy for 95% PTV in 33 fractions (1.82 Gy per fraction) and 66 Gy for gross tumor volume (GTV) in 33 fractions (2 Gy per fraction). Radiotherapy was administered five days a week using a Varian linear accelerator (6 MV X-ray). Concurrent chemotherapy consisted of cisplatin (40 mg weekly). At the last follow-up, the patient remained progression-free for over 20 months.

Throughout treatment, adverse events (AEs) were well-tolerated. No grade 3–4 AEs were observed, while grade 1–2 AEs included myelosuppression, nausea, vomiting, and radiation-induced skin injury.

**Figure 2:**
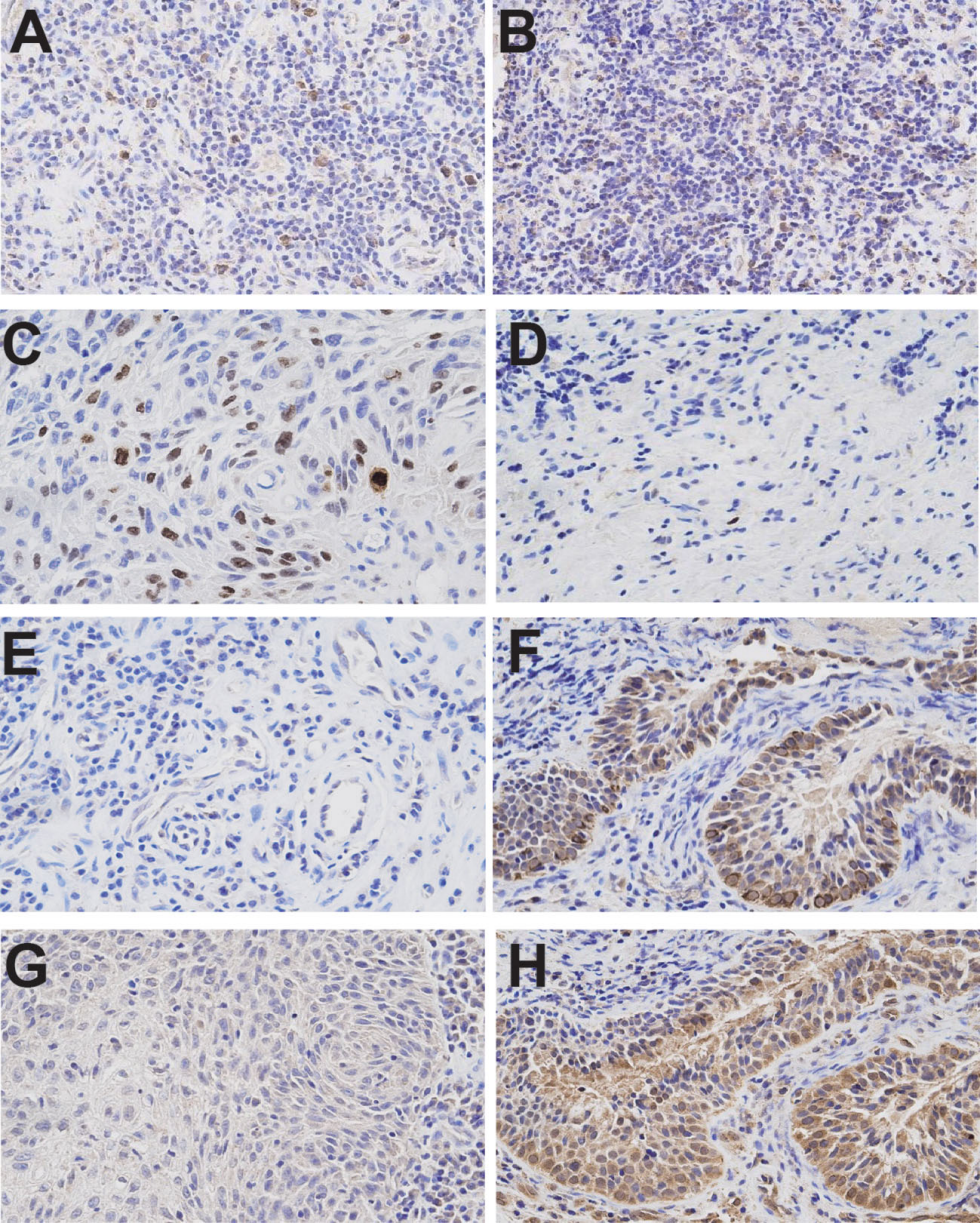
Immunohistochemical analyses of tumor samples before and after treatment. **(A)** PD-L1 immunohistochemistry (Ventana SP263 assay) from the initial diagnostic specimen. **(B)** PD-L1 immunohistochemistry from the final resection specimen. **(C)** Ki-67 immunohistochemistry from the initial diagnostic specimen. **(D)** Ki-67 immunohistochemistry from the final resection specimen. **(E)** IFN-γ expression in the initial diagnostic specimen. **(F)** IFN-γ expression in the final resection specimen. **(G)** CD8+ infiltrates in the initial diagnostic specimen. **(H)** CD8+ infiltrates in the final resection specimen. Images were taken at 400× magnification. Immunohistochemical analyses demonstrated a significant increase in PD-L1 and IFN-γ expression on tumor-infiltrating immune cells and CD8+ infiltrates, alongside a reduction in Ki-67 expression.

**Figure 3:**
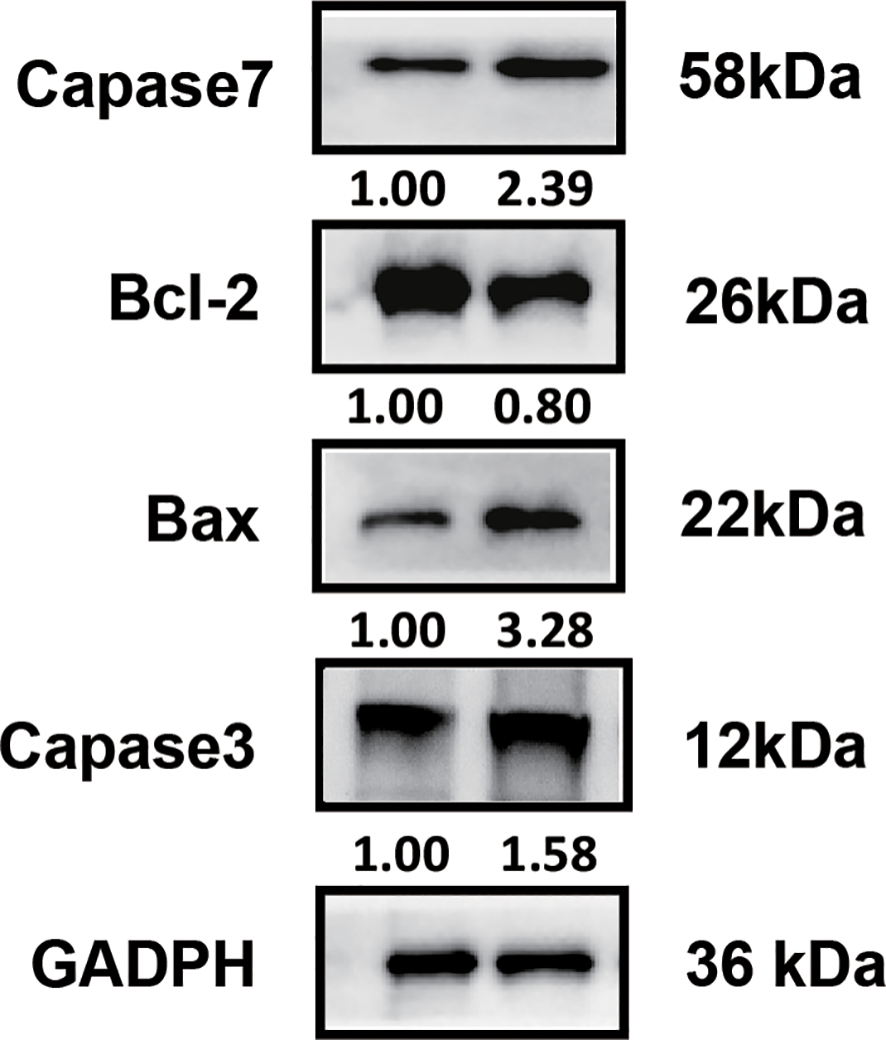
Western blot analysis of apoptosis-associated molecules. Elevated expression levels of pro-apoptotic molecules (Caspase-7, Caspase-3, Bax) and reduced expression of the anti-apoptotic molecule Bcl-2 were observed in post-treatment tumor samples.

To investigate the therapeutic mechanism, we assessed molecular markers of proliferation and apoptosis in baseline and post-treatment tumor specimens. Immunohistochemical analysis revealed a significant increase in PD-L1 and IFN-γ expression in tumor-infiltrating immune cells, along with elevated CD8+ cell infiltration and a reduction in Ki-67 expression (**Figure 2**). Western blot analysis demonstrated increased expression of pro-apoptotic proteins Caspase-7, Caspase-3, and Bax, accompanied by decreased expression of the anti-apoptotic protein Bcl-2 (**Figure 3**).

## Discussion

Neoadjuvant immunotherapy has demonstrated promising safety and tolerability profiles in HNSCC patients, alongside significant tumor shrinkage effects (14–17). In recent years, clinical studies investigating combinations of neoadjuvant immunotherapy with chemotherapy, radiotherapy, and targeted therapies in HNSCC have shown broad application potential for earlier-stage disease. Approximately 75% of HNSCC patients treated with neoadjuvant immunotherapy have achieved partial pathological responses, and preliminary data suggest that partial or major pathological responses may correlate with improved clinical outcomes (18).

HNSCC can be broadly classified into conventional types, which are predominantly associated with environmental factors such as tobacco and alcohol, and human papillomavirus (HPV)-related subtypes (19). Prognosis varies based on HPV status; patients with p16-positive HNSCC generally have better outcomes, while those with p16-negative HNSCC face poorer prognoses (20). For patients with p16-negative HNSCC and tumor invasion into adjacent organs, achieving R0 resection through surgery can be particularly challenging. Chemoradiotherapy, although effective, is associated with significant toxicities, such as myelosuppression, mucositis, and radiation-induced injuries, which can impair patient quality of life. These challenges emphasize the importance of exploring alternative strategies, such as neoadjuvant immunotherapy, to improve clinical outcomes while minimizing treatment-related morbidities.

Currently, five primary neoadjuvant strategies for HNSCC have been described: definitive immunotherapy plus concurrent chemoradiotherapy, definitive immunotherapy plus radiotherapy, neoadjuvant immunotherapy combined with radiotherapy, neoadjuvant immunotherapy with chemotherapy, and immunotherapy alone (21). In this case, a neoadjuvant regimen combining nivolumab with albumin-bound paclitaxel and carboplatin achieved a major pathological response (MPR), highlighting the potential efficacy of this approach.

Moreover, the patient and his family expressed a strong desire for organ preservation due to the tumor’s location and associated symptoms, including headache, diplopia, and restricted eye movement. This perspective significantly influenced the choice of treatment, favoring a less invasive yet effective neoadjuvant immunochemotherapy regimen. Post-treatment, the patient reported significant symptom relief and expressed satisfaction with the treatment outcome.

The identification of predictive biomarkers for immunotherapy in HNSCC remains a critical area of research. While PD-L1 positivity, high tumor mutational burden (TMB), and the presence of CD8+ lymphocytes are currently recognized as promising markers, further exploration is needed (22–25). Achieving pCR appears more likely with neoadjuvant stereotactic body radiotherapy (SBRT), where pCR rates have been reported (26). For patients without SBRT, MPR rates range from 2.9% (20) to 31% (14). In this case, the patient treated with nivolumab in combination with chemotherapy came close to achieving pCR. These findings suggest that combining neoadjuvant immunotherapy with other modalities could provide additional benefits to HNSCC patients (27).

Our case revealed several key biological changes following treatment. Postoperative analyses showed increased PD-L1 expression, elevated IFN-γ levels on tumor-infiltrating immune cells, and a significant increase in CD8+ lymphocyte infiltration, coupled with decreased Ki-67 expression. Western blot analyses further demonstrated upregulation of pro-apoptotic molecules, including Caspase-7, Caspase-3, and Bax, along with downregulation of anti-apoptotic Bcl-2, suggesting enhanced tumor cell apoptosis as a key mechanism driving therapeutic efficacy.

Despite these promising results, this case study has inherent limitations. As a single case, the findings may not be generalizable to the broader HNSCC population. Moreover, the challenges associated with surgery and chemoradiotherapy, such as high morbidity and incomplete pathological responses, further highlight the need for novel therapeutic strategies. Mechanistic analyses in this study suggested enhanced tumor cell apoptosis as a potential mode of action, providing a foundation for future investigations. Larger cohort studies are warranted to validate the clinical benefits of neoadjuvant immunotherapy combined with chemotherapy and to explore its broader applicability in HNSCC.

## Conclusion

In summary, this case highlights the successful application of neoadjuvant nivolumab combined with chemotherapy in an HNSCC patient, achieving significant tumor regression and organ preservation. Post-treatment analyses revealed increased PD-L1 expression, enhanced CD8+ lymphocyte infiltration, and decreased Ki-67 expression, indicating that the combination of immunotherapy and chemotherapy promotes tumor cell apoptosis and reshapes the immune microenvironment. These findings support the potential of neoadjuvant immunotherapy to improve clinical outcomes for HNSCC patients. However, this study has inherent limitations, as it is based on a single case, which limits the generalizability of the findings. Future larger cohort studies are needed to validate these results, optimize treatment regimens, and identify biomarkers for predicting therapeutic response. Establishing robust evidence will be critical for integrating neoadjuvant immunotherapy into standard clinical practice for HNSCC.

## Data Availability

The raw data supporting the conclusions of this article will be made available by the authors, without undue reservation.
